# Aberrant expression and potential therapeutic target of lysophosphatidic acid receptor 3 in triple-negative breast cancers

**DOI:** 10.1007/s10238-014-0306-5

**Published:** 2014-09-11

**Authors:** Kai Sun, Hui Cai, Xiaoyi Duan, Ya Yang, Min Li, Jingkun Qu, Xu Zhang, Jiansheng Wang

**Affiliations:** 1 The Second Department of Thoracic Surgery, The First Affiliated Hospital of Xi’an Jiaotong University, 277 West Yanta Road, Xi’an, 710061 Shaanxi China; 2 Department of Anesthesia, The First Affiliated Hospital of Xi’an Jiaotong University, Xi’an, Shaanxi China; 3 Department of Nuclear Medicine, The First Affiliated Hospital of Xi’an Jiaotong University, 277 West Yanta Road, Xi’an, 710061 Shaanxi China; 4 Department of Pathology, Lanzhou University Medical School, Lanzhou, Gansu China

**Keywords:** Lysophosphatidic acid receptor, Triple receptor-negative breast cancer, Aberrant expression, Therapeutic target

## Abstract

Triple receptor-negative breast cancers (TNBCs) generally have poor prognoses because of the loss of therapeutic targets. As lysophosphatidic acid (LPA) receptor signaling has been shown to affect breast cancer initiation and progression, we try to evaluate the potential roles of LPA receptors in TNBCs. We examined mRNA and protein expressions of LPA receptors 1-3, using quantitative real-time PCR and immunohistochemical analyses in normal (*n* = 37), benign disease (*n* = 55), and breast cancer tissues (*n* = 82). Carcinomas expressed higher levels of LPA_2_ and LPA_3_ mRNAs (0.17 ± 0.070 and 0.05 ± 0.023, respectively) than did normal breast tissue (0.13 ± 0.072 and 0.02 ± 0.002, respectively). Enhanced immunohistochemical staining for LPA_2_ and LPA_3_ protein was also consistently observed in carcinomas. The LPA_3_ overexpression was associated with lymph node metastases, and absence of estrogen receptor, progesterone receptors, and human epidermal growth factor receptor 2 expression. TNBC tissues and cell lines showed the highest LPA_3_ expression compared with luminal-type A and B breast cancers. Suppression of LPA_3_ by shRNA did not influence cell growth in breast cancer cells. However, the migration and invasion of TNBC cells were significantly inhibited by LPA_3_-shRNA or inhibitor, which had no or less effect on normal and non-TNBC breast cells. In conclusion, our data indicated that the expression of LPA receptor 3 was increased in human TNBCs and is associated with tumor metastatic ability, and this implies that LPA_3_ is a potential therapeutic target for the treatment of TNBCs.

## Introduction

Breast cancer is the most commonly diagnosed cancer, and the leading cause of cancer-related deaths, in women worldwide [1]. Cases are usually classified by their expression of estrogen receptors (ER) progesterone receptors (PR), and human epidermal growth factor-2 receptors (HER2), which together predict treatment response and prognosis [2]. Although hormone receptor (HR)^+^ breast cancers have many effective treatment options, fewer targeted therapies are available for triple receptor-negative breast cancers (TNBCs). Currently, some progress has been made in classifying TNBCs into several distinct subtypes using gene expression profiling analyses, and some kinases and agents were identified as potential druggable targets [3, 4], but the therapeutic implications are yet to be elucidated [5]. Thus, characterization of novel molecular biomarkers is critically required for the treatment of TNBCs.

Lysophosphatidic acid (LPA) receptors are specific G protein-coupled receptors binding with LPA, which mediates a variety of biological processes, such as cell proliferation, migration, invasion and differentiation [6]. At least six LPA receptors (LPA_1–6_) are currently identified, and their emerging roles in tumorigenesis have been demonstrated both in vitro and in vivo [7]. In breast tissue, LPA_1_ and LPA_2_ are broadly expressed in either normal or abnormal mammary epithelial cells, whereas expression levels of LPA_3–6_ are more restricted or undetectable, which may account for the various biological effects of LPA [8–10]. Overexpression of LPA_1_ and LPA_2_ was readily observed in breast cancers with redundant mediation functions in multiple endogenous LPA responses, including cancer cell growth, metastasis, angiogenesis, and chemoresistance [8, 11–13]. In contrast, less is known about the role of LPA_3_ in breast cancer initiation and progression. Previous published data showed that LPA_3_ was higher expression in poorly differentiated breast cancers than well-differentiated tumors [14, 15], which suggests that LPA_3_ contributes to breast cancer progression.

Although the LPA receptors have been shown to affect breast cancer initiation and progression, the exact expression patterns and functions in TNBCs have not been fully examined. In the present study, we characterized the expression of LPA_1–3_ in human normal, benign, and malignant breast tissues and cell lines, and analyzed correlations with clinical and pathological findings to highlight the possible roles of LPA receptors in the development and treatment of TNBCs.

## Materials and methods

### Patients

Specimens of normal breast (*n* = 37), mammaries with benign disease (*n* = 55), and breast cancer (*n* = 82) were collected from the First Affiliated Hospital of Xi’an Jiaotong University. This study was approved by the IRB of Xi’an Jiaotong University School of Medicine. All tissues were pathologically examined. Written informed consent forms were obtained from all subjects, and all clinical investigation had been conducted according to the principles expressed in the Declaration of Helsinki.

### RNA isolation and quantitative real-time PCR

Tissues or cells were lysed in the Qiagen RLT lysis buffer (Qiagen, USA). RNA was extracted with an RNeasy mini kit (Qiagen, USA) and reverse transcribed by M-MLV reverse transcriptase (Invitrogen, USA). Quantitative real-time PCR was performed on a Bio-Rad iQ5 Real-Time PCR Detection System (Bio-Rad Laboratories, USA) with a SYBR Green I Master Mix (TAKARA, Japan). PCRs were performed in triplicate, and the relative gene expression was calculated against GAPDH. Primer pairs used in this study were as follows: GAPDH: F, 5′-GAAGGTGAAGGTCGGAGT-3′/R, 5′-GAAGATGGTGATGGGATTTC-3′; LPA_1_: F, 5′-AATCGAGAGGCACATTACGG-3′/R, 5′-GTTGAAAATGGCCCAGAAGA-3′; LPA_2_: F, 5′-TTGTCTTCCTGCTCATGGTG-3′/R, 5′-TCAGCATCTCGGCAAGAGTA-3′; LPA_3_: F, 5′-TGCTCATTTTGCTTGTCTGG-3′/R, 5′-GCCATACATGTCCTCGTCCT-3′.

### Immunohistochemistry (IHC) analysis

Formalin-fixed paraffin-embedded sections (5 µm thick) of the normal breast, breast with benign diseases, and breast cancers were analyzed by IHC with the primary LPA_1–3_ antibody (1:100) and a biotin-conjugated secondary antibody. For IHC quantification, the sections were analyzed using Nikon TE2000-s microscope (Melville, USA). Four randomly selected areas were photographed at 40× magnification using a QimageRetiga 2000R camera (Surrey, Canada). The integral optical density (IOD) of immunoreactivity was calculated using the Image-Pro Plus image analysis software (Media Cybernetics, USA).

### Cell lines and culture

The MCF-10A and MCF-7 cells were obtained from Sagene Inc., (Guangzhou, China), and the MCF-12A, T47D, MDA-MB-231, and MDA-MB-157 cells were obtained from ATCC (Manassas, USA). All cell lines were maintained in a humidified atmosphere at 37 °C with 5 % CO_2_. MCF-10A, MCF-12A, and MCF-7 cells were cultured in DMEM with glutamine, 10 % FBS (Gibco, USA), and 100 μg/mL penicillin/streptomycin (P/S). T47D were cultured in RPMI1640 with glutamine, 10 % FBS (Gibco), and 100 μg/mL P/S. MDA-MB-231 and MDA-MB-157 cultured in Leibovitz’s L-15 Medium (ATCC, USA) with 10 % FBS (Gibco) and 100 μg/mL P/S.

### Western blot analyses

Western blot analyses were conducted using standard procedures, and proteins were detected using primary antibodies and fluorescent secondary antibodies (IRDye800CW-conjugated or IRDye680-conjugated anti-species IgG, Li-Cor Biosciences, Lincoln, NE, USA). The fluorescent signals were captured on an Odyssey Infrared Imaging System (Li-Cor Biosciences) with both 700- and 800-nm channels. Boxes were manually placed around each band of interest, and the software returned near-infrared fluorescent values of raw intensity with background subtraction (Odyssey 3.0 analytical software, Li-Cor Biosciences).

### shRNA transfection

Six-well plates were seeded with 5 × 10^4^ cells/well in 2 mL media 24 h before transfection; cells were 80–90 % confluent at transfection. Cells were transfected with LPA_3_ shRNA (100 pmol/well, Santa Cruz Biotechnology, USA) using Lipofectamine 2000 Reagent (Life Technologies, USA) according to the manufacturer’s instruction. After 48 h of transfection, cells were selected using puromycin for 2 weeks. Stable transductants were pooled.

### MTT assays

Cells were seeded at a density of 5 × 10^3^ cells/well in 96-well plates at a final volume of 180 µL in incubation, at 37 °C, with 5 % CO_2_. After different time incubation, 20 µL of 5 mg/mL solution of MTT (Sigma, MO, USA) in PBS was added to each well, and plates were then incubated for 4 h at 37 °C. Reaction crystals were then solubilized in 100 % dimethylsulfoxide (Sigma) 20 µL/well and shaken for 15 min. Absorbance of each well was measured on a multidetection microplate reader (BMG LABTECH, USA) at a wavelength of 570 nm.

### Cell migration and invasion Assays

Migration and invasion assays were conducted using transwell plates with 8-μm pore size membranes (Corning Inc., USA) as described previously [16]. After incubation for 4 h (for migration assays) or 24 h (for invasion assays), cells remaining in the upper side of the filter were removed with cotton swabs. The cells attached on the lower surface were fixed and stained using crystal violet and washed with water. Cells were counted with five high power fields per membrane, and results were presented as the mean number of cells migrated per field per membrane. All experiments were conducted in triplicate.

### Statistical analyses

Continuous variables were summarized as means with standard deviations (SD) across the healthy control, benign disease, and cancer groups. One-way ANOVA was used to test the overall difference, and Student’s *t* test was used to test the pairwise difference across disease statuses. Correlation between two different groups was tested by Pearson’s correlation coefficient. *P* < 0.05 was considered significant. All analyses were performed using SPSS software version 19.0 (IBM, USA).

## Results

### Expression patterns of LPA_1–3_ in breast tissues

**Fig. 1:**
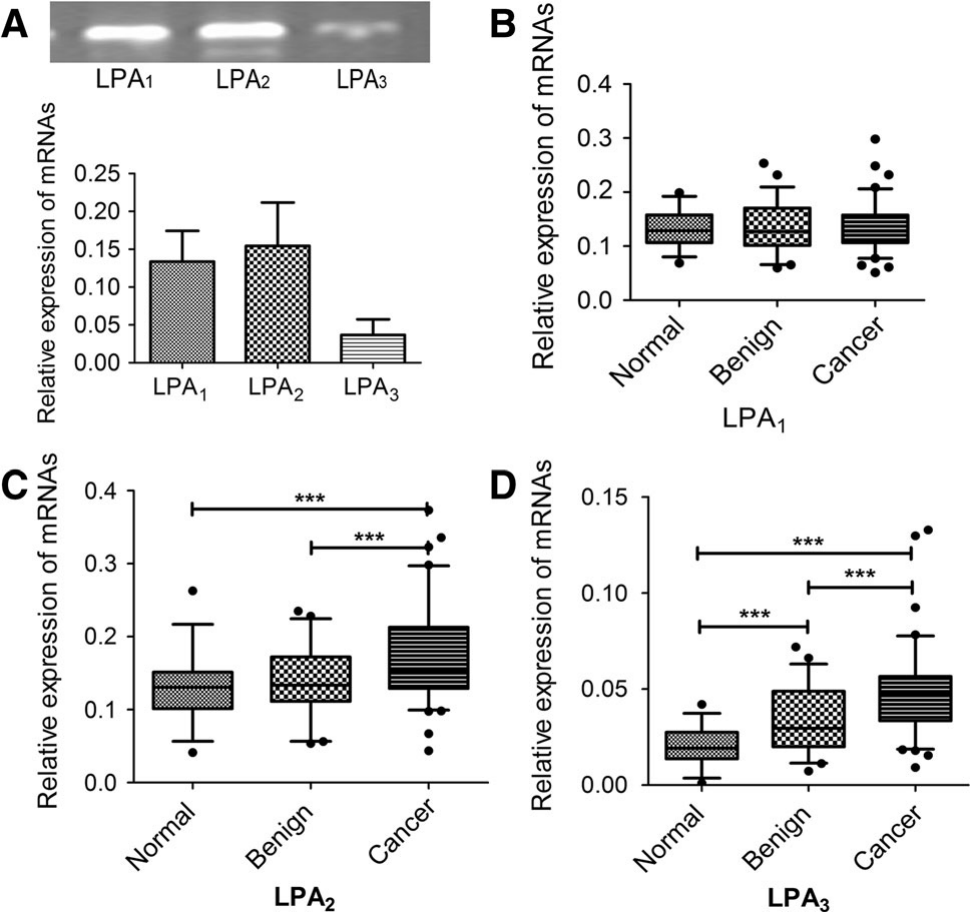
mRNA expression of LPA receptor 1-3 in breast tissues. **a** To determine whether the samples expressed LPA receptors, quantitative real-time PCR was performed by LPA_1_, LPA_2_, and LPA_3_ primers. The relative gene expression was calculated against GAPDH. **b** The relative LPA_1_ mRNA expression in normal breast epithelium, mammary with benign disease, and malignant tissues. **c** The relative LPA_2_ mRNA expression in normal breast epithelium, mammary with benign disease, and malignant tissues. **d** The relative LPA_3_ mRNA expression in normal breast epithelium, mammary with benign disease, and malignant tissues. ***P < 0.001

We evaluated mRNA expression of LPA_1–3_ in normal, benign, and malignant breast epithelium; mRNA levels were quantified against *GAPDH*. As shown in Fig. 1a, breast tissues predominantly expressed LPA_1_ and LPA_2_, whereas LPA_3_ expression was weakly but detectable in all specimens. Similar levels of LPA_1_ mRNA were detected in normal, benign, and carcinoma tissues (0.11 ± 0.058 vs. 0.13 ± 0.044 vs. 0.13 ± 0.034, *P* = 0.789; Fig. 1b). However, LPA_2_ mRNA levels in breast cancers were significantly higher than that in normal tissue (0.17 ± 0.070 vs. 0.13 ± 0.072, *P* = 0.0002; Fig. 1c). Although low levels of LPA_3_ were observed in all breast tissues, the cancer tissues exhibited a greater expression of LPA_3_ than did normal (0.05 ± 0.023 vs. 0.02 ± 0.002, *P* < 0.001) or benign-disease tissues (0.05 ± 0.023 vs. 0.03 ± 0.002, *P* < 0.001) (Fig. 1d). Notably, LPA_3_ expression was also greater in benign-disease tissue than in normal tissue (0.03 ± 0.002 vs. 0.02 ± 0.002, *P* = 0.009; Fig. 1d).

**Fig. 2:**
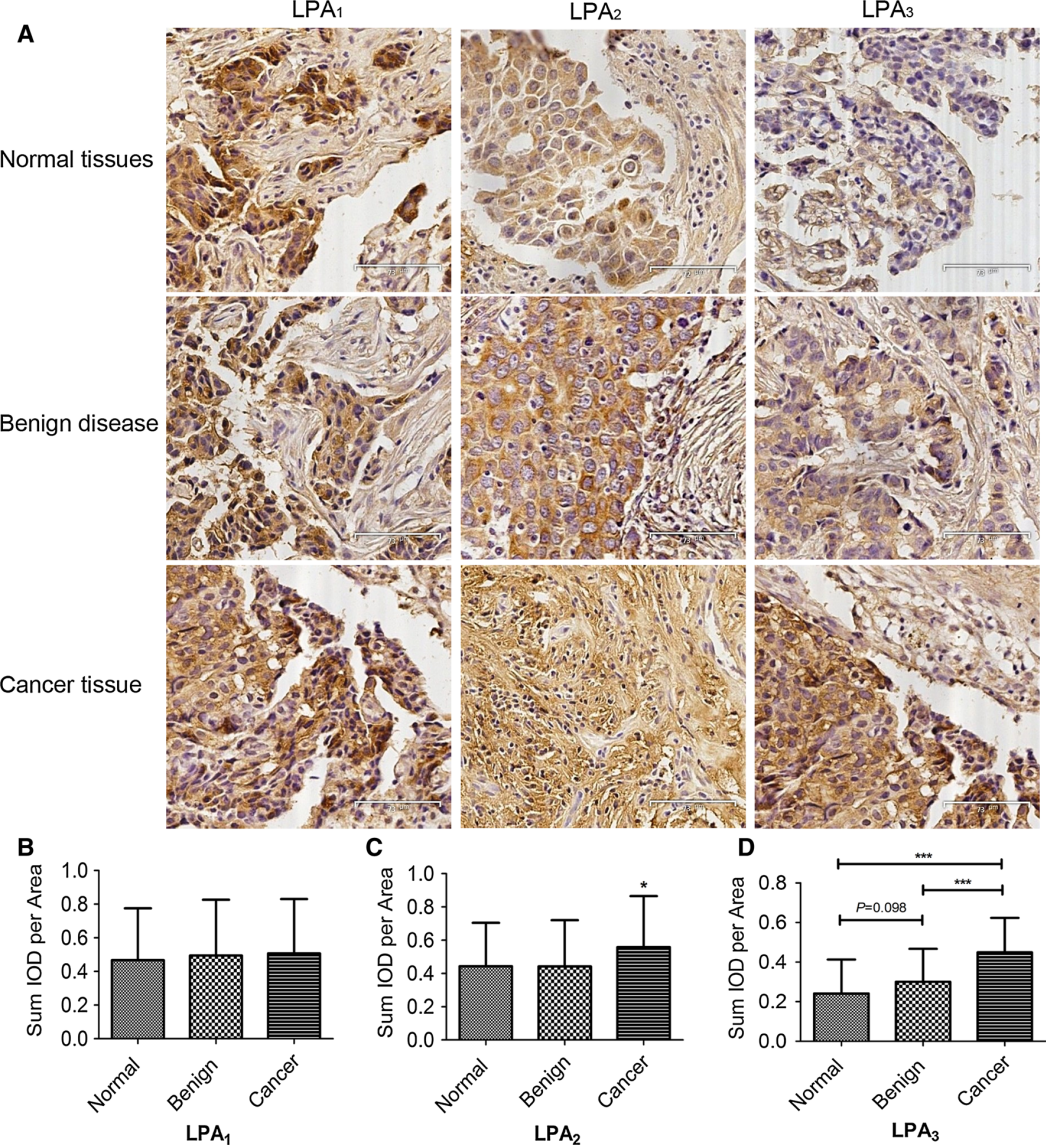
Protein levels of LPA receptor 1-3 in different breast tissues. **a** Immunostains for LPA receptor 1-3 in normal, benign disease and malignant breast tissue. **b**–**d** Quantification of immunostains for LPA receptor 1-3 by IOD analysis. *P < 0.05; ***P < 0.001

We also immunohistochemically evaluated expression of LPA receptor proteins in the same specimens (Fig. 2a). LPA_1–3_ was detectable in the cell membrane and cytoplasm in most specimens (113/119 of LPA_1_, 116/119 of LPA_2_, and 110/119 of LPA_3_). As with the mRNA expression, enhanced staining for LPA_2_ and LPA_3_ protein was clearly detected in carcinomas in comparison with normal epithelium or benign-disease tissues (Fig. 2c, d), whereas LPA_1_ expression did not differ significantly between different groups (Fig. 2b). Protein immunoreactivity significant correlated with relative mRNA expression (*r* = 0.592, *P* < 0.001).

### Relationship between LPA_1–3_ mRNA expression and clinical parameters in breast cancer patients

Relationships between LPA_1–3_ mRNA expression and clinical or pathological findings in 82 cases are presented in Table 1. LPA_1_ expression did not correlate with any clinical parameters. Higher expression of LPA_2_ was seen in postmenopausal patients (*P* < 0.05). The higher-stage tumors tended to express less LPA_2_, but not significantly (*P* = 0.095). The expression of LPA_3_ was associated with hormonal receptor status and lymph node metastases. ER^−^, PR^−^, or Her2^−^ tumors were more likely to express excess LPA_3_ than positive ones. Moreover, patients with lymph node metastases presented with higher LPA_3_ expression than patients without metastases (*P* < 0.05).

**Table 1:** Relationship between LPA receptors expression and clinical parameters of breast cancer

Clinical parameters	*N* (%)	LPA_1_	LPA_2_	LPA_3_
Age (years)				
<50	41 (50.0)	0.134 ± 0.039	0.173 ± 0.064	0.043 ± 0.020
≥50	41 (50.0)	0.133 ± 0.045	0.172 ± 0.065	0.051 ± 0.022
Stage				
I	14 (17.0)	0.134 ± 0.047	0.184 ± 0.059	0.047 ± 0.030
II	13 (15.9)	0.141 ± 0.042	0.178 ± 0.058	0.048 ± 0.014
III	20 (24.4)	0.127 ± 0.028	0.173 ± 0.069	0.041 ± 0.016
IV	35 (42.7)	0.134 ± 0.047	0.167 ± 0.066	0.050 ± 0.022
Grade				
1	29 (35.4)	0.134 ± 0.036	0.177 ± 0.060	0.040 ± 0.016
2	37 (45.1)	0.136 ± 0.048	0.164 ± 0.071	0.050 ± 0.026
3	16 (19.5)	0.125 ± 0.048	0.184 ± 0.070	0.051 ± 0.017
Tumor size				
≤2.0 cm	30 (36.6)	0.124 ± 0.036	0.173 ± 0.063	0.049 ± 0.023
2.0–5.0 cm	36 (43.9)	0.143 ± 0.046	0.177 ± 0.068	0.049 ± 0.020
>5.0 cm	16 (19.5)	0.130 ± 0.039	0.163 ± 0.058	0.037 ± 0.019
Menopausal status				
Premenopausal	32 (39.0)	0.133 ± 0.043	0.154 ± 0.046*	0.043 ± 0.022
Postmenopausal	50 (61.0)	0.134 ± 0.041	0.185 ± 0.071	0.049 ± 0.021
ER status				
Negative	37 (45.1)	0.133 ± 0.045	0.169 ± 0.060	0.057 ± 0.023***
Positive	45 (54.9)	0.134 ± 0.039	0.176 ± 0.067	0.038 ± 0.015
PR status				
Negative	43 (52.4)	0.132 ± 0.044	0.180 ± 0.072	0.055 ± 0.023***
Positive	39 (47.6)	0.135 ± 0.040	0.166 ± 0.054	0.037 ± 0.014
Her2 status				
Negative	55 (67.1)	0.135 ± 0.043	0.164 ± 0.055	0.051 ± 0.023**
Positive	27 (32.9)	0.131 ± 0.041	0.191 ± 0.077	0.038 ± 0.013
Nodal metastasis				
Negative	37 (45.1)	0.132 ± 0.042	0.177 ± 0.064	0.034 ± 0.013***
Positive	45 (54.9)	0.135 ± 0.042	0.170 ± 0.064	0.057 ± 0.021

* *P* < 0.05; ** *P* < 0.01; *** *P* < 0.001

### Higher expression of LPA_3_ in TNBC tissues and cell lines

**Fig. 3:**
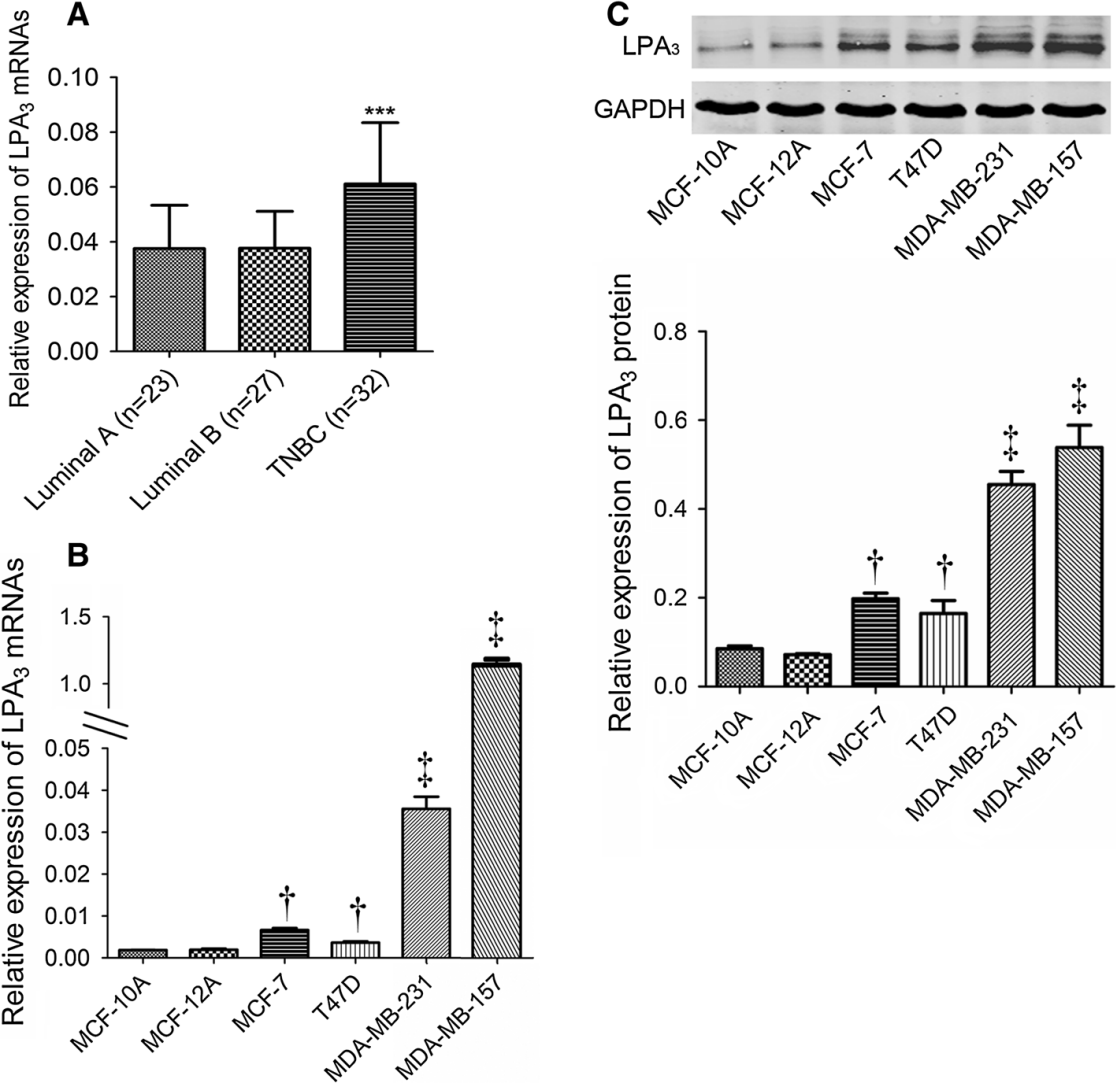
High expression of LPA_3_ in TNBCs. **a** The relative LPA3 mRNA expression in breast cancer tissues from luminal A, luminal B and TNBC patients. **b** The relative LPA_3_ mRNA expression in six different breast cell lines was determined by quantitative real-time PCR. The results are presented as the mean ± SD against GAPDH obtained in three independent experiments. **c** Western blots were used to detect protein levels of LPA_3_ in six breast cell lines. Quantification of protein was presented as the mean ± SD of fluorescent values obtained in three independent experiments. ^†^Compared to normal mammary cells, *P* < 0.05; ^‡^compared to normal mammary cells or non-TNBC cells, *P* < 0.001

As LPA_3_ expression in carcinomas strongly correlated with HR status, we subsequently analyzed the distributions of LPA_3_ among different tumor immunophenotypes. Breast cancer patients were classified as luminal A, luminal B, and TNBC, based on their expression of ER, PR, Her2, and ki-67 [17]. LPA_3_ expression differed significantly among tumors with different immunophenotypes (*P* < 0.001; Fig. 3a). The highest LPA_3_ protein level was demonstrated in the TNBCs whereas similar expressions were found between luminal A and luminal B carcinomas.

To confirm the expression profiles of LPA_3_ in TNBCs, we further detected the mRNA and protein levels of LPA_3_ in normal mammary epithelial cells and breast cancer cell lines with different molecular phenotypes. As expected, breast cancer cell lines (MCF-7, T47D, MDA-MB-231, and MDA-MB-157) expressed more LPA_3_ than normal immortal cells (MCF-10A and MCF-12A), and the highest expression of LPA_3_ was detected in the TNBC cells (MDA-MB-231 and MDA-MB-157) (Fig. 3b, c).

### Inhibition of LPA_3_ by shRNA decreased migration and invasion of TNBC cells

**Fig. 4:**
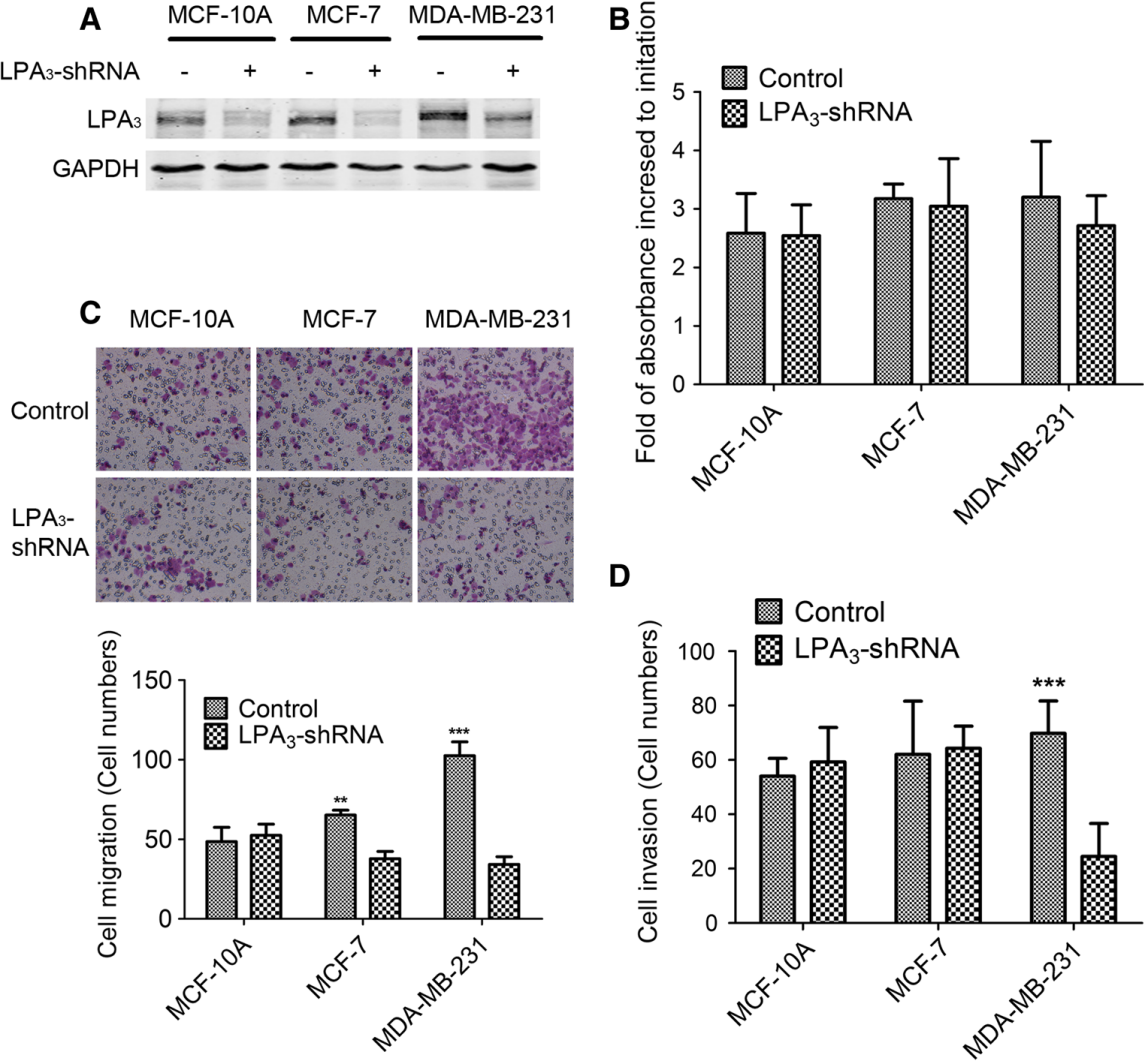
Inhibition of LPA_3_ decreased migration and invasion of TNBC cells. **a** Expression of LPA_3_ was decreased by shRNA. MCF-10A, MCF-7, and MDA-MB-231 cells were transfected with control and LPA_3_ shRNA. Forty-eight hours later, cell lysates were analyzed by Western blots with anti-LPA_3_ antibody. **b** The effect of LPA_3_ on breast cancer cells growth, as measured using the MTT assay. The results are presented as the mean ± SD of fold increased to initiation obtained in 3 independent experiments. **c**, **d** Cell transwell assays were conducted to investigate the role of LPA_3_ on breast cancer cells migration (**c**) and invasion (**d**). The results are presented as the mean ± SD of cell number obtained in three independent experiments. **P < 0.01; ***P < 0.001

To further analyze the role of LPA_3_ in breast tumorigenesis, we conducted cell proliferation, migration, and invasion assays of LPA_3_- and control-shRNA-transfected breast epithelial cells, including normal immortal cells MCF-10A, luminal cells MCF-7, and TNBC cells MDA-MB-231. LPA_3_ was effectively down-regulated by shRNA in all three cell lines (Fig. 4a). Cell proliferation tested by MTT showed that suppression of LPA_3_ did not influence cell growth in all three cell lines (Fig. 4b). However, cells with LPA_3_-shRNA migrated significantly less than controls in MDA-MB-231 cells (Fig. 4c). Although LPA_3_-shRNA also reduced migration of MCF-7 cells, the inhibitory capacity was weaker than in MDA-MB-231 cells (Fig. 4c). We also assessed the effect of LPA_3_ knockdown on cellular invasion and revealed LPA_3_ loss significantly decreased invasion of MDA-MB-231 cells, but had less or no effect on MCF-10A and MCF-7 cells (Fig. 4d).

### Ki16425 dose-dependently suppressed migration and invasion of TNBC cells

**Fig. 5:**
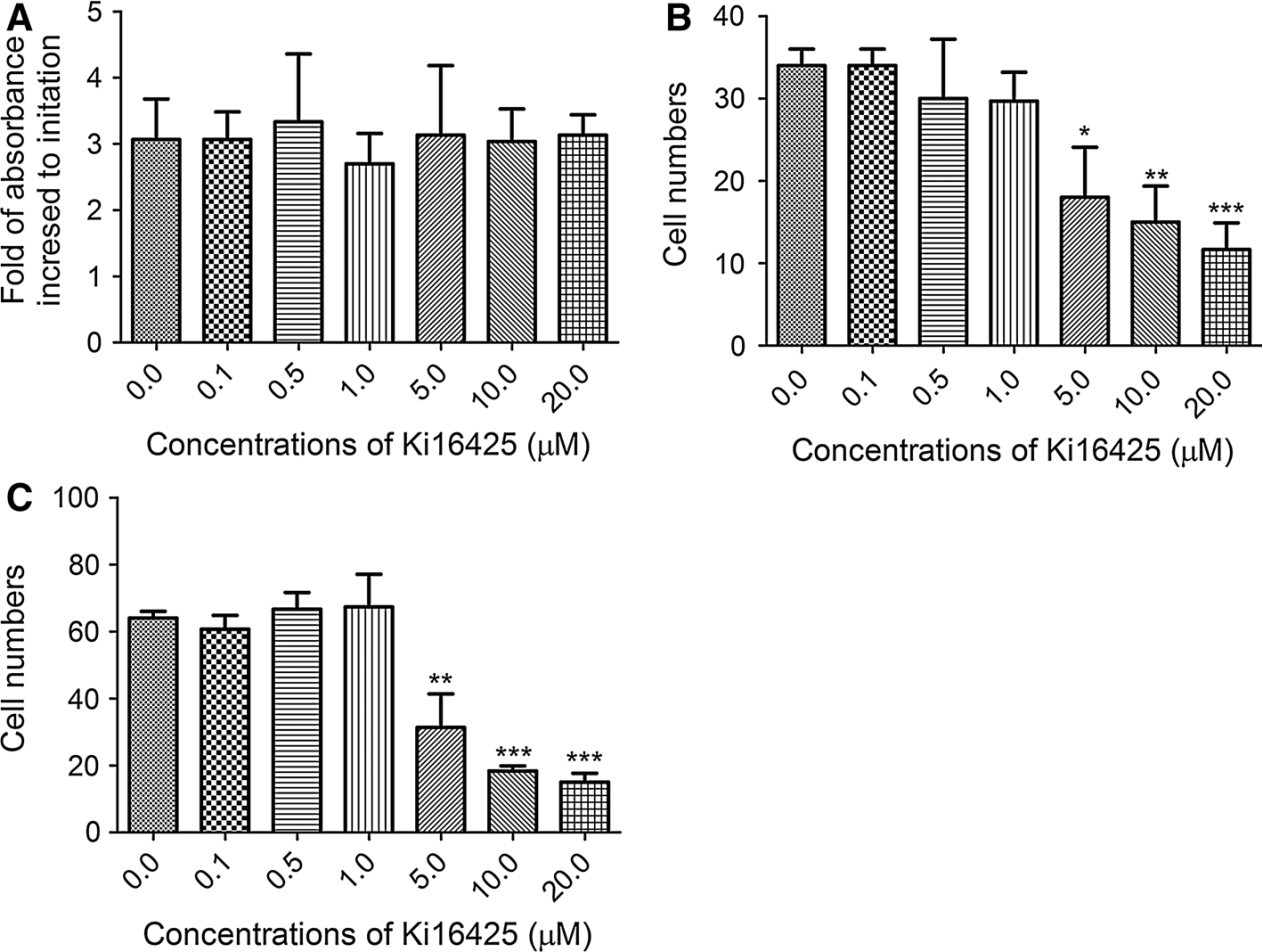
Inactivated LPA_3_ by Ki16425 suppressed migration and invasion of TNBC cells. **a** MDA-MB-231 cells were treated with indicated concentrations of Ki16428 for 1 h, and then cell viability was measured using the MTT assay. The results are presented as the mean ± SD of fold increased to initiation obtained in 3 independent experiments. **b**, **c** Migration (**b**) and invasion (**c**) of MDA-MB-231 cells were inhibited by ki16425. MDA-MB-231 cells were pretreated with indicated concentrations of ki16425 for 1 h and then transferred to collagen- or matrigel-coated transwell chambers for migration and invasion experiments, respectively. *P < 0.05; **P < 0.01; ***P < 0.001

We used Ki16425, an antagonist for LPA_1_ and LPA_3_, to confirm the critical roles of LPA_3_ in TNBC cells. We first showed that pre-treating MDA-MB-231 cells with Ki16425 did not influence cell viability (Fig. 5a). We then evaluated the effects of Ki16425 on migration and invasion of TNBC cells, using transwell assays. As shown in Fig. 5b, c, Ki16425 suppressed migration and invasion of MDA-MB-231 cells in a dose-dependent manner.

## Discussion

LPA receptors are expressed by normal mammary epithelial cells, with aberrant expression occurring during breast cancer initiation and progression [18]. In the present study, we found abnormal expression of LPA receptors in mammary carcinomas, and that LPA_2_ and LPA_3_ expression was enhanced in breast cancer compared with normal breast and benign-disease tissues, although the expression level of LPA_1_ was not significantly different between each subgroup. Particularly, we also showed significantly increased LPA_3_ expression in the TNBCs compared with other immunophenotype tumors. Subsequently, function analysis revealed that inhibition of LPA_3_ by shRNA or antagonist dramatically suppressed the migration and invasion ability of TNBC cells, but had no or less effect on normal or luminal-type cancer cells, which suggests a role for LPA_3_ in the pathophysiology of TNBCs.

Expression and function of LPA_1_ in the breast cancer have been studied extensively. Overexpression of LPA_1_ is readily observed in breast cancer cells [19]. Manipulation of LPA_1_ level or function could alter the survival and metastatic ability of breast cancer cells both in vitro and in vivo [11, 12, 14, 20, 21]. However, in our present research, LPA_1_ expression levels did not differ significantly between normal and malignant breast tissues. This inconsistency may result from tumor heterogeneity. In breast cancer, LPA_1_ is expressed in many cancer cell lines, but at various levels. It is most likely that LPA_1_ higher expressed in more aggressive cell lines, such as MDA-MB-231, and lower in less aggressive breast cancer cells, such as MCF-7 [10, 13, 20, 22, 23]. In accord with cell lines, LPA_1_ mRNA was significantly more abundant in advanced stages of breast cancer compared with noninvasive stage breast tumors [24]. Moreover, accumulating evidence indicates that the LPA_1_ contributes to the metastatic capability of breast cancers. Higher LPA_1_ expression is significantly related to positive node and bone metastases [11, 12], which implies that LPA_1_ affects breast cancer progression. However, some clinical studies found no major expression pattern for the LPA_1_ between breast cancer patients and normal controls [8, 25], which suggests that LPA_1_ has no role in breast cancer initiation.

From an evolutionary perspective, tumors can be as genetically and epigenetically heterogeneous cell populations, although most human tumors are monoclonal outgrowths descending from single progenitor cells [26, 27]. As tumor progression, genetic and epigenetic alterations occur in progeny cells. However, changes in LPA_1_ expression as they affect breast cancer initiation and progression are barely understood and require additional exploration.

Although the expression and functions of LPA_2_ have been the subject of fewer studies, increased LPA_2_ expression has been reported in invasive breast carcinoma [8, 14]. Transgenic mice that overexpress LPA_2_ showed higher incidence of mammary tumors with early onset than mice that overexpress LPA_1_, which implicates LPA_2_ in the initiation of breast cancer [14]. In vitro studies, LPA_2_ has been verified to regulate LPA-induced breast cancer cells proliferation and migration through Erk or RhoA pathway [23, 28]. Recently, a literature also reported LPA_2_ involved in LPA-induced IL-6 and IL-8 expression, which promoted colony formation and cell survival of TNBCs [29]. Together with our findings that LPA_2_ is more highly expressed in breast cancer patients, these combined data validate LPA_2_ as a potential therapeutic target for drug development and evaluation in breast cancer.

As with LPA_2_, little is known about the expression patterns of LPA_3_ in breast cancers. Until recently, Nikolay et al. indicated that LPA_3_ was higher expressed in human breast cancers, and most interesting LPA_3_ overexpression was associated with absence of ER and PR [30], which suggests a function of LPA_3_ in HR^−^ carcinomas. Our studies confirmed that LPA_3_ was overexpression in ER^−^/PR^−^/Her2^−^ tumor cells and tissues when compared with normal breast epithelium and luminal-type cancers. Inhibition of LPA_3_ significantly decreased migration and invasion of TNBC cells but did not affect other immunotype breast cancers, reflecting on dominant metastatic roles of LPA_3_ in TNBCs.

Cancer metastasis is a complex biological event of multiple steps, one of which is epithelial to mesenchymal transition (EMT), a prelude to increased cellular motility and plasticity, which thereby enables cellular invasion [31, 32]. Initial evidence for a possible role of LPA and its receptors in EMT was derived from experiments in hepatocellular carcinoma and ovarian cancer by showing a proline-rich tyrosine kinase 2 (PYK2) or periostin (alias osteoblast-specific factor-2)-induced EMT, upon LPA treatment [33, 34]. In the breast cancer, Jahn et al. [35] demonstrated that LPA_1_ is up-regulated in cells that underwent EMT and consequently led to an increased responsiveness to LPA after EMT. These results imply that the LPA receptors contribute to cell EMT. The roles of different LPA receptors in EMT clearly merit wider investigation.

As a receptor for LPA, LPA_3_ can promote cancer progression. However, the downstream pathways of LPA_3_ are rarely elucidated. Currently, evidences indicated that Yes-associated protein (YAP), a transcriptional factor of Hippo pathway, is a critical downstream mediatio of LPA_3_ in ovarian cancer [36]. Thus, we invested the expression correlation between LPA_3_ and YAP protein and found that tumors with overexpression of LPA_3_ exhibited week YAP staining (data not shown). As YAP functions as a breast tumor suppressor [37], LPA_3_-YAP pathway may involved in initiation and progression of breast cancers. Interesting, another study revealed that loss of YAP expression is associated with estrogen and PR negativity in breast carcinomas [38]. Besides, in transgenic mice model, the ER, PR, and Her2 were significantly decreased in LPA_3_ overexpression mice when compared to wild-type mice [14]. All together, LPA_3_, YAP, and hormonal receptors may interact in TNBC pathophysiology. However, the exact mechanism is unclear and requires further study.
